# Prevalence and Its Associated Factors of Anemia Among Pregnant Women Attending Antenatal Care in Health Institutions of Hargeisa, Somaliland

**DOI:** 10.1155/anem/5067972

**Published:** 2026-05-25

**Authors:** Naima Abdikarim, Haftu Asmerom, Zerihun Ataro, Ephrem Tefera Solomon

**Affiliations:** ^1^ Department of Nursing and Midwifery, Edna Aden University Hospital, Hargeisa, Somaliland; ^2^ School of Medical Laboratory Sciences, College of Health and Medical Sciences, Haramaya University, Harar, Ethiopia, haramaya.edu.et; ^3^ School of Medical Laboratory Sciences, College of Health Sciences, Arsi University, Asella, Ethiopia, arsiun.edu.et

**Keywords:** anemia, association factors, Hargeisa, pregnant women, Somaliland

## Abstract

**Background:**

Anemia is one of the most common worldwide public health problems related to pregnancy. However, there is a scarcity of evidence regarding anemia among pregnant women in Somaliland, particularly in Hargeisa city. Therefore, this study aimed to assess the prevalence and its associated factors of anemia among pregnant women attending antenatal care in health institutions of Hargeisa, Somaliland.

**Method:**

A cross‐sectional study was carried out by involving 402 pregnant women in health institutions of Hargeisa between August 30 and September 30, 2023. Data were collected by four certified midwives using a structured face‐to‐face interview questionnaire, while four experienced laboratory professionals collected the necessary laboratory samples. A probability proportion to size sampling method was used, followed by simple random selection to select the study participants. The hemoglobin level was measured using the HemoCue 301 System. Data were entered into EpiData Version 4.6 and exported to SPSS Version 26 for analysis. A logistic regression model was fitted to determine the factors associated with anemia. Variables with a *p* value less than 0.25 in the bivariable analysis were considered for the multivariable logistic regression model. Statistical significance was set at *p* < 0.05, and results were reported as adjusted odds ratios with 95% confidence intervals.

**Results:**

Of the 402 pregnant women, 56.2% (95% CI: 51%–61%) of the participants were anemic, of which 20.4% were mild anemia, 32.6% were moderate anemia, and 3.2% were severe anemia. Pregnant women who had no ANC follow‐up before the current visit (AOR = 3.42, 95% CI = 1.91, 6.18), pregnant women who did not use iron supplementation (AOR = 3.18, 95% CI = 1.85, 5.48), pregnant women who were in the third trimester (AOR = 3.29, 95% CI = 1.69, 6.42), pregnant women infected with intestinal parasitosis (AOR = 5.12, 95% CI = 1.08, 24.15), and pregnant women who were positive for malaria (AOR = 7.71, 95% CI = 1.81, 32.54) were all significantly associated with maternal anemia.

**Conclusions:**

The prevalence of anemia among pregnant women in Hargeisa is a significant public health concern, with more than half of the study participants affected.

## 1. Introduction

Anemia is a disorder that occurs when there are fewer red blood cells (RBCs) or a lower quantity of hemoglobin (Hb) [1]. It happens at every stage of life, but the risk is higher in pregnancy because of the increased physiological demand, iron need, and blood loss [2, 3]. The World Health Organization (WHO) and the Centers for Disease Control and Prevention (CDC) define maternal anemia as a Hb level below 11 g/dL [4, 5], making it one of the most common pregnancy‐related problems [6]. Accordingly, Hb between 10.0 and 10.9 g/dL is considered mild anemia, Hb between 7.0 and 9.9 g/dL is considered moderate anemia, and Hb below 7.0 g/dL is considered severe anemia [7].

Although there are several forms of anemia, including iron deficiency anemia (IDA), hemolytic anemia, aplastic anemia, and vitamin B12 deficiency anemia [8], IDA (about 75%) and megaloblastic anemia from poor diets and lack of prenatal folate supplements are the most prevalent forms of anemia during pregnancy [9]. The physiologic drop in the Hb level is explained by these anemias, which cause disproportionate alterations in the RBC by increasing plasma volume at around six weeks of gestational age in pregnant women [10].

More than 115,000 maternal morbidities and 591,000 postnatal mortalities occur worldwide each year due to anemia, a public health issue that affects 1.62 billion individuals worldwide, of whom 56 million were pregnant [11]. In 2019, the WHO reported that 29.9% of women of reproductive age worldwide, equivalent to more than half a billion women between the ages of 15 and 49, were anemic, with a prevalence of 36.5% in pregnant women [12]. A total of 17.2 million (57.1%) cases are shared by Africa [13], with Egypt reporting a high frequency of anemia (45%) [14]. In sub‐Saharan Africa, its prevalence varied from 35.6% to 57% [15, 16]. A multilevel study of recent East African demographic and health surveys found that 41.82% of pregnant women had anemia, with significant variation across individual nations, ranging from 23.36% in Rwanda to 57.10% in Tanzania [17]. In 2019, the World Bank Group reported that 48.7% of pregnant Somalian women had anemia [18].

In addition to being a significant contributor to poor pregnancy outcomes like low birth weight (LBW), prematurity, intrauterine growth restriction, perinatal and neonatal mortality, low Apgar score, and poor cognitive and motor development [19, 20], anemia during pregnancy promotes the alteration of placental angiogenesis, which limits the fetus’s access to nutrients and results in fetal growth retardation and low weight at birth [21]. Furthermore, as anemia was responsible for more than 20% of maternal deaths, it is associated with a significant risk of maternal morbidities, including abortions, antepartum and postpartum hemorrhages, preeclampsia, protracted labor, and maternal mortality [22, 23]. Growth retardation of the infant and obstructed labor due to cephalopelvic disproportion are consequences of IDA. These conditions are linked to high rates of maternal and perinatal morbidity and mortality, preterm deliveries, stillbirths, neonatal deaths, and the risk of developing severe anemia because the mother’s physiological needs and the developing fetus’s nutritional needs compete [24].

Although some local studies were conducted in Somaliland [25, 26], the prevalence of anemia was not reported in some studies, and associated factors were also not clearly articulated in the other studies. Therefore, this study aimed to determine the prevalence and its associated factors of anemia among pregnant women attending antenatal care (ANC) in health institutions of Hargeisa, Somaliland.

## 2. Materials and Methods

### 2.1. Study Design, Area, and Period

Hargeisa town, the capital and largest city of Somaliland, was the site of an institution‐based cross‐sectional study. Hargeisa town is 1,334 m above sea level and is situated in the Moroodi Jeex area, which is in a valley of the Galgodon highlands. An estimated 1.2 million people live there, and 360,000 of them are of reproductive age on average. There are 20 health centers, 12 private hospitals, and one governmental hospital in this town. Two hospitals (Hargeisa General Hospital and Edna Adan Hospital) and two health centers (Somaliland Family Health Association (SOFHA) and Abdi Eidan Health Center) were the sites of the study. These health institutions provide comprehensive integrated sexual‐related health services, including antenatal, postnatal, delivery, family planning, and pediatric services, and provide all mother and child health basic programs [27]. The study was conducted at these four selected health institutions from August 30 to September 30, 2023.

### 2.2. Population, Inclusion, and Exclusion Criteria

All pregnant women who visited ANC at the selected health institutions and consented to participate in the study were included in the study population. However, pregnant women who were unable to talk due to severe illness were excluded from the study.

### 2.3. Sample Size Determination and Sampling Technique

A single population proportion formula was used to calculate the sample size for the prevalence of anemia, taking into account the following assumptions: a 95% confidence level, a 5% margin of error, and the proportion of anemia among pregnant women in Ethiopia (23.3%) [23]. This provided the intended sample size of 275. The sample size for the associated factors was calculated using Open Epi online software under the following assumptions: 95% confidence level, 80% power, equal unexposed‐to‐exposed ratio (1:1), proportion of anemia in the third trimester of gestational age (19.8%), and first trimester of gestational age (9.0%) [28]. This provided a sample size of 368.

**FIGURE 1:**
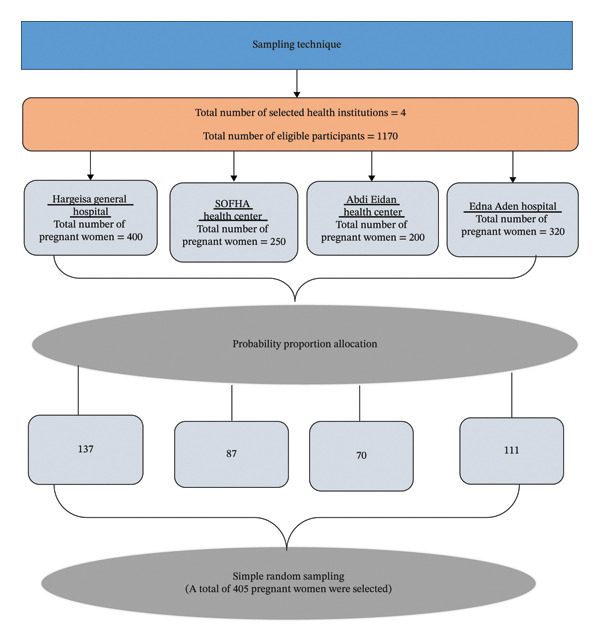
Schematic presentation of sampling procedure among pregnant women attending antenatal care in health institutions of Hargeisa, Somaliland, from August 30 to September 30, 2023. Abbreviation: SOFHA: Somaliland Family Health Association.

For this study, the sample size determined for associated factors of anemia (368) was utilized as it was larger than the sample size determined for anemia prevalence. After adding 10% for non‐respondents, the ultimate sample size was 405 study participants. The average number of pregnant women who visited ANC in the previous month before the study was conducted in all selected health institutions was 1,170 (720 pregnant women in hospitals and 450 pregnant women in health centers). Sampling frames were established, and both simple random sampling and probability proportion to size allocation were used to estimate the number of study participants to be included in the study for each health institution (Figure 1).

## 3. Data Collection and Measurements

### 3.1. Data Were Collected Using the Following

Face‐to‐face interview: four certified midwives conducted in‐person interviews using a structured questionnaire that was modified from earlier literature [29–31]. Sociodemographic details, mother’s obstetric history, nutrition‐related variables, and medical history were all gathered using the questionnaire.

Dietary diversity assessment: The minimum dietary diversity score for women (MDD‐W) was calculated to assess the micronutrient adequacy of the participants’ diets. Pregnant women were asked about their consumption of foods from the following ten pre‐established food groups over the previous 24 h: (1) grains, white roots and tubers, and plantains; (2) pulses (beans, peas, and lentils); (3) nuts and seeds; (4) dairy; (5) meat, poultry, and fish; (6) eggs; (7) dark green leafy vegetables; (8) vitamin A–rich fruits and vegetables; (9) other vegetables; and (10) other fruits [32]. Participants who consumed at least five out of these 10 food groups were categorized as having met the minimum dietary diversity, while those consuming fewer than five groups were categorized as having inadequate dietary diversity [33].

Anthropometric measurement: A mid‐upper arm circumference (MUAC) tape was used to measure the MUAC in centimeters. The measurement of the right upper arm was taken at the halfway between the elbow and shoulder tips. As a result, pregnant women with MUACs ≥ 23 cm were considered normal, whereas those with MUACs < 23 cm were deemed undernourished [34].

### 3.2. Laboratory Sample Collection and Examinations

Four experienced laboratory professionals gathered all of the laboratory samples needed for this investigation. Thus, a portable heme analyzer (HemoCue 301 Hb; HemoCue analyzer, Ängelholm, Sweden) was used to test Hb levels. Each participant had 1 μL of capillary blood drawn, which was then put into a microcuvette, cleaned of extra blood from the exterior of the microcuvette tip, and put into the cuvette holder of the Hb measurement instrument in g/dL [35]. Hb levels were corrected for altitude as advised by the WHO because it was more than 1,000 m above sea level. As a result, with an average elevation of 1,334 m above sea level in Hargeisa town, the adjusted Hb concentration was computed as follows: Hb = −0.032 × (altitude in meters × 0.0033) + 0.022 × (altitude in meters × 0.0033)^2^. Lastly, to generate adjusted Hb values, 0.3 g/dL was deducted from an individual‐measured Hb in g/dL [36].

Additionally, two and one drop of capillary blood were taken from each pregnant woman for the preparation of thick and thin blood films, respectively. Next, in accordance with WHO recommendations, thick and thin blood films were made [37]. Absolute methanol was used to fix the thin films, and the 10% Giemsa stain was applied for 10 minutes to both thick and thin smears. Following drying, the slides were examined under a microscope with 100x objectives to look at malaria parasites. On the other hand, all study subjects had 20 mg of freshly passed feces collected using a clean plastic cup and a wooden applicator. The feces were then placed on a slide and emulsified with a drop of physiological saline (0.85%). The preparation was covered with a cover slip before being viewed under a microscope with 10x and 40x objectives to check the presence of intestinal parasites.

### 3.3. Operational Definitions

Anemia: A condition where the Hb level in the body is less than 11 g/dL [5], and it is classified as mild anemia (Hb 10.0 g/dL–10.9 g/dL), moderate anemia (Hb 7.0–9.9 g/dL), and severe anemia (Hb < 7.0 g/dL) [7].

Food diversity: the dietary diversity score (DDS) was defined as the number of food groups consumed by the mother in the previous days [38].

### 3.4. Data Processing and Analysis

Following data collection, the data were edited and cleaned, and each questionnaire was coded and double‐entered into Epi‐Data Version 4.6 and exported into Statistical Package for Social Sciences (SPSS) Version 26 for analysis. Tables, percentages, frequencies, and figures were used to describe categorical variables. Bivariate logistic regression analysis was employed, and the crude odds ratio (COR) with 95% CI was computed to analyze the relationship between each independent variable and the outcome variable. For the multivariable logistic regression analysis, variables with *p* values less than 0.25 were considered. The Hosmer and Lemeshow test was used to assess the model’s goodness of fit, and the results showed that the final model suited the data well. By calculating the adjusted odds ratio (AOR) with a 95% CI, the final model was run to control the confounding variables and identify the associated factors. Multicollinearity among the independent variables was assessed using the variance inflation factor (VIF), with a VIF value of less than 10 considered indicative of the absence of significant collinearity. A *p* value of less than 0.05 was considered statistically significant.

### 3.5. Data Quality Control

To ensure uniformity, the questionnaire was first created in English, translated into the local language (Af‐Somali), and then translated back into English. The questionnaire was pretested on 5% of the sample size for three days outside the selected health institutions one week before the actual data collection, and any necessary corrections were made. Every stage of the whole data collection procedure was closely monitored. Every laboratory test and nutritional evaluation was carried out following SOPs. The laboratory test was conducted using the SOP principle, and the production reagents, their expiration dates, and their appropriate storage were examined to guarantee quality. As soon as the sample was taken from the study participants, it was processed. Supervisors reviewed filled questionnaires for completeness, and any errors, ambiguities, incompleteness, or other issues were resolved.

### 3.6. Ethical Considerations

The study was carried out after ethical approval was obtained from the Institutional Health Research Ethics Review Committee (IHRERC), College of Health and Medical Sciences, Haramaya University, with reference number (IHRERC‐/162/2023). A letter of permission was obtained from the district administrative Health Bureau and from all selected health institutions. All study participants provided informed, voluntary, written, and signed consent prior to data collection. Confidentiality was maintained, and any information and findings obtained during the study were kept confidential. Additionally, anemic pregnant women were also linked to the respective health professionals for better management and care.

## 4. Results

### 4.1. Sociodemographic Characteristics of Pregnant Women

A total of 402 pregnant women took part in the study, resulting in a response rate of 99%. Refusal to participate was the reason for the non‐responses. The mean age of the participants was 27.9 years (standard deviation [SD] = 6.2), which ranged between 15 and 45 years. The majority of the study participants were married: 362 (90.0%) and 299 (74.4%) were housewives. In terms of educational status, 159 (39.6%) respondents were unable to read and write, whereas 29 (7.2%) respondents were enrolled in college or above. One hundred twelve (27.9%) of the respondents had an income of ≥ 400 dollars, and 17 (4.2%) had an income of < 100 dollars, respectively (Table 1).

**TABLE 1:** Sociodemographic characteristics of pregnant women attending antenatal care in health institutions of Hargeisa, Somaliland, from August 30 to September 30, 2023 (*n* = 402).

Variables	Variable category	Number (%)
Age	15–20	71 (17.7)
	21–25	106 (26.3)
	26–30	90 (22.4)
	31–35	95 (23.6)
	36–45	40 (10.0)

Marital Status	Married	362 (90.0)
	Divorced	28 (7.0)
	Widowed	12 (3.0)

Occupation Status	Student	20 (5.0)
	Housewife	299 (74.4)
	Government employee	40 (10.0)
	Self‐employed	32 (8.0)
	NGO	11 (2.6)

Educational Status	Unable to read and write	159 (39.6)
	Able to read and write	80 (19.9)
	Elementary school	73 (18.2)
	Secondary school	61 (15.1)
	College or above	29 (7.2)

Residence	Rural	62 (15.4)
	Urban	340 (84.6)

Family Income (in dollars)	< 100	17 (4.2)
	100–199	80 (19.9)
	200–299	111 (27.6)
	300–399	82 (20.4)
	≥ 400	112 (27.9)

Abbreviation: NGO; nongovernmental organization.

### 4.2. Obstetric‐Related Characteristics of Pregnant Women

Less than half of the study participants, 198 (49.3%), had visited ANC before; hence, about 120 (29.9%) got it 2–5 times during their current pregnancy. However, almost more than half, 204 (50.7%), of pregnant women did not have ANC follow‐up, and the reason for not visiting ANC follow‐up was mainly lack of money (72 [17.9%]). All pregnant women, 198 (49.3%), who had had ANC follow‐up took iron supplementation. More than half of the pregnant women, 209 (52%), were in the third trimester. Three hundred fifteen (78.4%) of the pregnant women reported that they had not experienced an antepartum hemorrhage (APH) in the past. Regarding gravida, the majority, 326 (81.1%), of pregnant women were multigravida. From those who had a history of multigravida, 179 (44.5%) had a birth interval of ≤ 2 years between the last and the current pregnancy, and 245 (60.9%) of pregnant women were multipara (Table 2).

**TABLE 2:** Obstetric‐related characteristics of pregnant women attending antenatal care in health institutions of Hargeisa, Somaliland, from August 30 to September 30, 2023 (*n* = 402).

Variables	Variable category	Number (%)
ANC follow‐up before the current visit	Yes	198 (49.3)
	No	204 (50.7)

Number of ANC visits (*n* = 198)	< 2	44 (22.2)
	2–5	120 (60.6)
	> 5	34 (17.2)

Reasons for not visiting ANC (*n* = 204)	Lack of money	72 (35.3)
	Lack of awareness	65 (31.9)
	Long distance	56 (27.4)
	Others	11 (5.4)

Iron supplementation during current pregnancy	Yes	198 (49.3)
	No	204 (50.7)

Gravida	Prima gravida	76 (18.9)
	Multigravida	326 (81.1)

Birth interval for multigravida	≤ 2 years	179 (44.5)
	> 2 years	147 (36.6)

Parity	Nullipara	25 (6.2)
	Primipara	51 (12.7)
	Multipara	245 (60.9)
	Grand multipara	81 (20.2)

Gestational age	First trimester	82 (20.4)
	Second trimester	111 (27.6)
	Third trimester	209 (52)

History of APH	Yes	87 (21.6)
	No	315 (78.4)

*Note:* ANC; antenatal care, APH; antepartum hemorrhage.

### 4.3. Nutrition‐Related Factors of Pregnant Women

Even if the majority of pregnant women, 306 (76.1%), visited ANC, 44 (10.9%) pregnant women were undernourished. Regarding food habits, 118 (29.4%) pregnant women had the habit of eating food made from cereals and grains ≤ 2 times/week. One hundred twelve (27.9%) pregnant women had the habit of eating food made from legumes and nuts more than once per day. Majority of women, 125 (31.1%), had the habit of eating fruits after a meal more than once per day. Majority of the study subjects, 192 (47.8%), had the habit of eating animal products (beef, goat, chicken, or other kinds of organ meat) 1–2 times per month (Table 3).

**TABLE 3:** Nutrition‐related factors of pregnant women attending antenatal care in health institutions of Hargeisa, Somaliland, from August 30 to September 30, 2023 (*n* = 402).

Variables	Variable category	Number (%)
MUAC	Undernourished	44 (10.9)
	Normal	358 (89.1)

Eating food made from cereals and grains	≤ 2 times/week	118 (29.4)
	3–4/week	72 (17.9)
	Once/day	110 (27.4)
	> 1/day	102 (25.3)

Eating food made from legumes and nuts	≤ 2 times/week	100 (24.9)
	3–4/week	88 (21.8)
	Once/day	102 (25.4)
	> 1/day	112 (27.9)

Eating fruits after a meal	≤ 2 weeks	113 (28.1)
	3–4/week	85 (21.1)
	1/day	79 (19.7
	> 1/day	125 (31.1)

Eating animal products (beef, goat, chicken, or other kinds of organ meat)	Never	9 (2.2)
	1–2/month	192 (47.8)
	1–2/week	135 (33.6)
	≥ 3/week	66 (16.4)

## 5. Laboratory Findings and Other Medical Problem‐Related Factors

Pregnant women who sought ANC had their Hb levels measured. As a result, 226 of them (56.2%) had Hb levels below 11 g/dL. According to the findings of the stool examination, about 26 (6.5%) of them tested positive for different intestinal parasites. About 8 (2%) of the pregnant women who tested positive for parasites had *Giardia lamblia*, followed by *Entamoeba histolytica* (6, 1.6%) and *Hymenolepis nana* (7, 1.7%). Thirty‐three (8.1%) pregnant women tested positive on the blood film examination. Furthermore, 68 (16.8%) of the study participants also had a history of medical problems, with hypertension, 28 (6.9%), found to be high (Table 4).

**TABLE 4:** Laboratory findings and other medical problems of pregnant women attending antenatal care in health institutions of Hargeisa, Somaliland, from August 30 to September 30, 2023 (*n* = 402).

Variables	Variable category	Number (%)
Hemoglobin	< 11 g/dL	226 (56.2)
	≥ 11 g/dL	176 (43.8)

Stool examination for intestinal parasite	Positive	26 (6.5)
	Negative	376 (93.5)

Type of intestinal parasite (*n* = 26)	*Giardia labia*	8 (2)
	*Hymenolepis nana*	7 (1.7)
	*Entamoeba histolytica*	6 (1.5)
	*Ascaris lumbricoides*	3 (0.7)
	Others	2 (0.5)

Blood film examination	Infected	26 (6.5)
	Noninfected	376 (93.5)

History of medical problems	Yes	68 (16.8)
	No	334 (83.2)

Type of medical problems	Hypertension	28 (6.9)
	Asthmatic	16 (4)
	Epilepsy	4 (1)
	Diabetic	4 (1)
	Others	6 (1.5)

### 5.1. Prevalence and Severity of Anemia Among Pregnant Women

**FIGURE 2:**
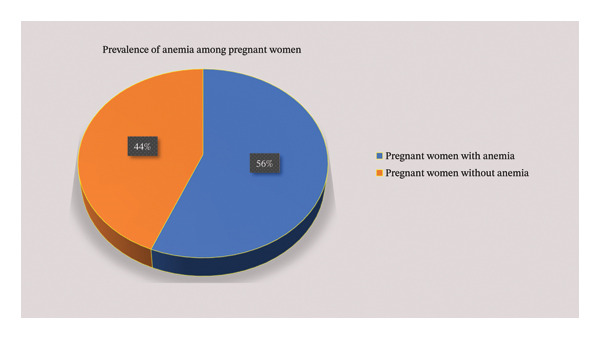
Prevalence of anemia among pregnant women attending antenatal care in health institutions of Hargeisa, Somaliland, from August 30 to September 30, 2023 (*n* = 402).

The mean Hb level of the study participants was 10.25 ± 1.78 g/dL with a range of 5.4–13.4 g/dL. The overall prevalence of anemia among pregnant women was 56.2% (95% CI: 51%–61%) (Figure 2).

**FIGURE 3:**
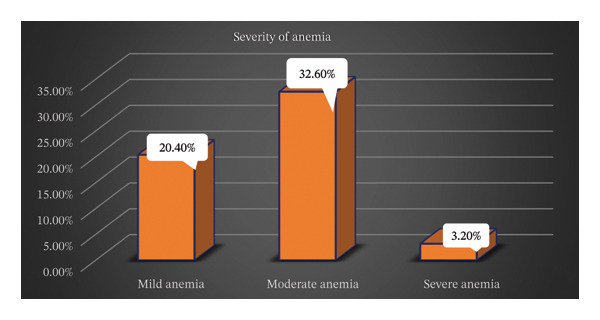
Severity of anemia among pregnant women attending antenatal care in health institutions of Hargeisa, Somaliland, from August 30 to September 30, 2023 (*n* = 402).

Out of this prevalence, 82 (20.4%) were mild anemia, 131 (32.6%) were moderate anemia, and 13 (3.2%) were severe anemia, respectively (Figure 3).

### 5.2. Factors Associated With Anemia Among Pregnant Women

According to the bivariable logistic regression analysis result, maternal age, residence, ANC follow‐up before the current visit, iron supplementation, parity, MUAC, eating food made from cereals and grains, stool examination, and blood film examination were significantly associated at a *p* value of < 0.25 and considered potential candidates for multivariable logistic regression analysis. In multivariable logistic regression, ANC follow‐up before the current visit, iron supplementation, gestational age, blood film examination, and stool examination were independently associated with greater odds for the presence of anemia at a *p* value of < 0.05.

Pregnant women who had no ANC follow‐up before the current visit were 3.42 times more likely (AOR = 3.42, 95% CI = 1.91, 6.18) to develop anemia than those who had. Pregnant women who did not use iron supplementation were 3.18 times more likely (AOR = 3.18, 95% CI = 1.85, 5.48) to develop anemia than their counterparts. Pregnant women who were in the third trimester of gestational age had 3.29 times higher odds (AOR = 3.29, 95% CI = 1.69, 6.42) of developing anemia compared to those who were in the first trimester. Pregnant women with intestinal parasitosis had 5.12 times more chance (AOR = 5.12, 95% CI = 1.08, 24.15) of developing anemia than their counterparts. Additionally, pregnant women who were positive for malaria were 7.71 times (AOR = 7.71, 95% CI = 1.81, 32.54) more at risk of anemia than those who were negative (Table 5).

**TABLE 5:** Factors associated with anemia among pregnant women attending antenatal care in health institutions of Hargeisa, Somaliland, from August 30 to September 30, 2023 (*n* = 402).

Variables	Category	Anemia status		95% CI	
		Yes, *N* (%)	No, *N* (%)	COR	AOR
Age in years	15–20	40 (56.3)	31 (43.7)	0.49 (0.21, 1.13)	0.61 (0.20, 1.82)
	21–25	45 (42.5)	61 (57.5)	0.28 (0.13, 0.62)	0.44 (0.16, 1.21)
	26–30	55 (61.1)	35 (38.9)	0.59 (0.26, 1.34)	1.24 (0.41, 3.88)
	31–35	57 (60.0)	38 (40.0)	0.57 (0.25, 1.27)	0.68 (0.26, 1.85)
	36–45	29 (72.5)	11 (27.5)	1	1

Residence	Rural	46 (74.2)	16 (25.8)	1	1
	Urban	180 (52.9)	160 (47.1)	0.39 (0.21, 0.72)	0.79 (0.35, 1.74)

Educational status	Unable to read/write	94 (59.1)	65 (40.9)	1.55 (0.70, 3.43)	1.68 (0.61, 4.65)
	Able to read/write	52 (65.0)	28 (35.0)	1.99 (0.84, 4.71)	1.34 (0.47, 3.92)
	Elementary School	39 (53.4)	34 (46.6)	1.23 (0.52, 2.91)	2.25 (0.76, 6.72)
	Secondary school	27 (44.3)	34 (55.7)	0.85 (0.35, 2.06)	1.41 (0.48, 4.35)
	College or above	14 (48.3)	15 (51.7)	1	1

Marital status	Married	200 (55.2)	162 (44.8)	0.41 (0.11, 1.54)	0.39 (0.08, 1.96)
	Divorced	17 (60.7)	11 (39.3)	0.51 (0.11, 2.33)	0.30 (0.05, 1.98)
	Widowed	9 (75.0)	3 (25.0)	1	1

ANC follow‐up before the current visit	Yes	83 (41.9)	115 (58.1)	1	1
	No	143 (70.1)	61 (29.9)	3.24 (2.15, 4.90)	3.42 (1.91, 6.18)

Iron supplementation	Yes	89 (44.9)	109 (55.1)	1	1
	No	137 (67.2)	67 (32.8)	2.50 (1.67, 3.57)	3.18 (1.85, 5.48)

Gestational age	1st trimester	34 (41.5)	48 (58.5)	1	1
	2nd trimester	53 (47.7)	58 (52.3)	1.29 (0.72, 2.29)	1.48 (0.71, 3.05)
	3rd trimester	139 (66.5)	70 (33.5)	2.80 (1.65, 4.73)	3.29 (1.69, 6.42)

Gravidity	Primigravida	43 (56.6)	33 (43.4)	1.01 (0.61, 1.68)	1.56 (0.72, 3.44)
	Multigravida	183 (56.1)	143 (43.9)	1	1

Parity	Nullipara	13 (52.0)	12 (48.0)	1	1
	Multipara	129 (52.7)	116 (47.3)	1.02 (0.45, 2.33)	1.29 (0.33, 5.12)
	Grand multipara	60 (74.1)	21 (25.9)	2.63 (1.04, 6.67)	2.31 (0.54, 10.15)
	Primipara	24 (47.1)	27 (52.9)	0.82 (0.31, 2.13)	1.35 (0.30, 6.22)

MUAC	Undernourished	33 (75.0)	11 (25.0)	2.56 (1.25, 5.23)	2.24 (0.89, 5.62)
	Normal	193 (53.9)	165 (46.1)	1	1

Eating food made from cereals and grains	≤ 2 times/week	56 (47.5)	62 (52.5)	0.51 (0.29, 0.88)	0.59 (0.23, 1.48)
	3–4/week	40 (55.6)	32 (44.4)	0.71 (0.38, 1.31)	2.11 (0.65, 7.14)
	Once/day	65 (59.1)	45 (40.9)	0.82 (0.47, 1.43)	2.38 (0.76, 7.41)
	> 1/day	65 (63.7)	37 (36.3)	1	1

Eating food made from legumes and nuts	≤ 2 times/week	47 (47.0)	53 (53.0)	0.61 (0.35, 1.06)	0.52 (0.19, 1.45)
	3–4/week	43 (48.9)	45 (51.1)	0.66 (0.37, 1.16)	0.63 (0.21, 1.89)
	Once/day	70 (68.6)	32 (31.4)	1.52 (0.86, 2.67)	1.08 (0.39, 2.98)
	> 1/day	66 (58.9)	46 (41.1)	1	1

Eating animal products (beef, goat, chicken, or other kinds of organ meat)	Never	5 (55.6)	4 (44.4)	1.41 (0.34, 5.72)	2.85 (0.31, 27.2)
	1–2/month	108 (56.3)	84 (43.7)	1.45 (0.82, 2.54)	1.93 (0.80, 4.68)
	1–2/week	82 (60.7)	53 (39.3)	1.74 (0.96, 3.16)	1.52 (0.55, 4.31)
	≥ 3/week	31 (47.0)	35 (53.0)	1	1

Stool examination	Positive	24 (92.3)	2 (7.7)	10.33 (2.40, 44.36)	5.12 (1.08, 24.15)
	Negative	202 (53.7)	174 (46.3)	1	1

Blood film	Infected	23 (88.5)	3 (11.5)	6.5 (1.92, 22.13)	7.71 (1.81, 32.54)
	Noninfected	203 (54.0)	173 (46.0)	1	1

*Note:* ANC: antenatal care.

Abbreviation: MUAC: mid‐upper arm circumference.

## 6. Discussion

The study’s findings examined the prevalence of anemia and its associated variables in pregnant women attending ANC in Hargeisa, Somaliland’s health institutions. Consequently, 56.2% of pregnant women had anemia, according to the results of the Hb test. The following factors were found to be associated with anemia among pregnant women who visited the ANC ward: pregnant women who were in the third trimester of gestation, pregnant women who were overweight, pregnant women with intestinal parasitosis, pregnant women who tested positive for malaria, pregnant women who had no ANC follow‐up prior to the current visit, and pregnant women who did not take iron supplements.

In this study, the overall prevalence of anemia among pregnant women who visited the ANC ward was 56.2%, of whom 20.4% had mild anemia, 32.6% had moderate anemia, and 3.2% had severe anemia. This study was concordant with the studies conducted in Bangladesh 58.9% [39], Libya 56.5% [40], Kenya 57% [41], and Ethiopia 56.8% [22, 28]. However, compared to earlier studies conducted in Yemen 93% [42], India 63% [43], Bangladesh 62.5% [30], and Somalia 84.3% [44], the current study findings showed a decreased prevalence of anemia and a higher prevalence of anemia than other similar studies conducted in Saudi Arabia 44% [45], Indonesia 40.7% [46], Ghana 50.8% [47], Nigeria 37.6% [48], and Sudan 22% [49]. Variations in the sample size, data collection time, Hb measurement technique, nutritional state, and sociodemographic characteristics of the participants may be the cause of this discrepancy.

Pregnant women who had no ANC follow‐up before the current visit were 3.42 times more likely (AOR = 3.42, 95% CI = 1.91, 6.18) to develop anemia than those who had. The result was in agreement with the studies conducted in Sudan [50], Somalia [14], and India [51]. According to earlier research, anemia is prevented by the benefits of ANC follow‐up, improving women’s empowerment, and maintaining a healthy maternal nutritional status [52]. Furthermore, women who started obtaining ANC treatments could interact with doctors more frequently and gain a better understanding of the advantages and disadvantages of taking iron supplements to prevent anemia during pregnancy [53].

Pregnant women who did not use iron supplementation were 3.18 times more likely (AOR = 3.18, 95% CI = 1.85, 5.48) to develop anemia than their counterparts. This finding was supported by previous studies conducted in Bangladesh [54], Tanzania [55], and Ethiopia [53]. Pregnant women who take iron‐containing supplements or a combination of iron and folate have been shown to enhance pregnancy outcomes and maternal anemia [56].

Pregnant women who were in the third trimester of gestational age had 3.29 times higher odds (AOR = 3.29, 95% CI = 1.69, 6.42) of developing anemia compared to those who were in the first trimester. This study was in agreement with studies conducted in Bangladesh [39], Eswatini [57], Ethiopia [58], and Somalia [14]. In the third trimester, the need for absorbed iron of 7.5 mg/day and the decrease in Hb concentration are explained by the greater maternal plasma volume increases (40%–50%) compared to red cell mass (20%–30%) [59].

Pregnant women who were positive for stool examination were 5.12 times more likely (AOR = 5.12, 95% CI = 1.08, 24.15) to be anemic compared to those who were negative. This finding was consistent with studies conducted in Ethiopia [60, 61]. The possible explanation is that intestinal colonization might lead to intestinal erosion, which can result in malnutrition in the patient [62]. In addition to causing gastrointestinal blood loss, this also depletes iron, folic acid, and vitamin B12, which eventually results in anemia [63].

Pregnant women who had a malaria attack were 7.71 times (AOR = 7.71, 95% CI = 1.81, 32.54) more at risk of anemia than those who did not have an attack. This finding was in line with studies conducted in Indonesia [64] and Ethiopia [31]. Pregnant women are more vulnerable to malaria because the placenta sequesters malaria parasites, preventing splenic clearance [65]. Maternal anemia can be caused by malaria in a variety of ways, such as excessive removal of nonparasitized erythrocytes, immunological death of parasitized RBCs, and reduced erythropoiesis due to bone marrow dysfunction [66].

### 6.1. Strengths and Limitations of the Study

The study’s strength is that it was laboratory‐based that included Hb measurements, stool examination, and blood film examination. However, this study included pregnant women who came to health institutions cannot be generalized to the community. A cross‐sectional study cannot show the temporal relation between identified associated factors and maternal anemia. Additionally, the use of dietary recall to assess minimum dietary diversity may be subject to recall bias, as participants might not perfectly remember or may inaccurately report all food groups consumed. Additionally, while this study adjusted for several known risk factors, the possibility of residual confounding remains due to unaddressed variables. Anemia prevalence through biochemical tests like serum ferritin and genetic predispositions of the study participants were also not assessed.

## 7. Conclusions

Of the examined pregnant women, more than half were found to be anemic, of which one‐fifth, one‐third, and close to one‐twentieth were found to be mild anemia, moderate anemia, and severe anemia, respectively. No ANC follow‐up before the current visit, no iron supplementation usage, third trimester of gestational age, positive for stool examination, and malaria infection showed a significant association between pregnant women who visited the ANC ward and anemia. Therefore, we would suggest that health institutions in Hargeisa pay special attention to pregnant women to encourage early antenatal attendance. There should be routine screening of anemia for all pregnant women, considering the factors like iron supplementation, taking appropriate nutritional foods during pregnancy, and preventing and controlling intestinal parasites and malaria infections. In the future, a cohort study utilizing sophisticated Hb testing, serum ferritin levels, and genetic predispositions should be conducted to proactively screen for anemia.

**Nomenclature**
ANC: Antenatal care
AOR: Adjusted odds ratio
APH: Antepartum hemorrhage
CDC: Centers for Disease Control and Prevention
CI: Confidence interval
COR: Crude odds ratio
DDS: Dietary diversity score
Hb: Hemoglobin
IDA: Iron deficiency anemia
IHRERC: Institutional Health Research Ethics Review Committee
LBW: Low birth weight
MDDS: Minimum dietary diversity score
MUAC: Mid‐upper arm circumference
RBC: Red blood cells
SD: Standard deviation
SOFHA: Somaliland Family Health Association
SOP: Standard operating procedure
WHO: World Health Organization

## Author Contributions

Naima Abdikarim, Haftu Asmerom, Ephrem Tefera Solomon, and Zerihun Ataro conceived the study, drafted the proposal, monitored data collection, and coordinated field work. Naima Abdikarim, Haftu Asmerom, Ephrem Tefera Solomon, and Zerihun Ataro carried out data analysis and interpretation of the findings and wrote the manuscript. Ephrem Tefera Solomon had full access to all of the data in this study and takes complete responsibility for the integrity of the data and the accuracy of the data analysis.

## Funding

The principal investigator, Naima Abdikarim, an M.Sc. Student in Tropical and Infectious Diseases at Haramaya University, was funded by the University with ID. No. SGS/0256/14. Hence, the authors want to declare that no funding was received for this study from external sources.

## Disclosure

All authors have read and approved the final version of the manuscript.

## Conflicts of Interest

The authors declare no conflicts of interest.

## Data Availability

The dataset generated and analyzed for this study can be obtained from the corresponding author upon reasonable request.

## References

[1] World Health Organization , Guideline on Haemoglobin Cutoffs to Define Anaemia in Individuals and Populations, 2024, World Health Organization, https://www.who.int. 38530913

[2] Mbule M. A. , Byaruhanga Y. B. , Kabahenda M. , and Lubowa A. , Determinants of Anaemia Among Pregnant Women in Rural Uganda, Rural and Remote Health. (2013) 13, no. 2, 1–15, 10.22605/rrh2259. 23679828

[3] McLean E. , Cogswell M. , Egli I. , Wojdyla D. , and de Benoist B. , Worldwide Prevalence of Anaemia, WHO Vitamin and Mineral Nutrition Information System, 1993-2005, Public Health Nutrition. (2009) 12, no. 4, 444–454, 10.1017/s1368980008002401, 2-s2.0-65449184945. 18498676

[4] Centers for Disease Control and Prevention , Anemia Among Pregnant Women Participating in the Special Supplemental Nutrition Program for Women, Infants, and children—United States, 2008–2018 2022, https://www.cdc.gov/mmwr/volumes/71/wr/mm7125a1.htm. 10.15585/mmwr.mm7125a1 35737575

[5] World Health Organization , Strategies to Prevent Anaemia: Recommendations from an Expert Group Consultation, 2016, World Health Organization.

[6] Haroon F. , Dhrolia M. F. , Qureshi R. , Imtiaz S. , and Ahmed A. , Frequency of Pregnancy-Related Complications Causing Acute Kidney Injury in Pregnant Patients at a Tertiary Care Hospital, Saudi Journal of Kidney Diseases and Transplantation. (2019) 30, no. 1, 194–201, 10.4103/1319-2442.252910. 30804281

[7] World Health Organization , Guideline: Daily Iron and Folic Acid Supplementation in Pregnant Women, 2012, World Health Organization. 23586119

[8] Jablonka A. , Wetzke M. , Sogkas G. et al., Prevalence and Types of Anemia in a Large Refugee Cohort in Western Europe in 2015, Journal of Immigrant and Minority Health. (2018) 20, no. 6, 1332–1338, 10.1007/s10903-018-0725-6, 2-s2.0-85044447304. 29582203

[9] Olayinka O. and &Ogunbode O. , Anaemia in Pregnancy, Contemporary Obstetrics and Gynecology for Developing Countries. (2021) 321–330.

[10] Soma-Pillay P. , Nelson-Piercy C. , Tolppanen H. , and Mebazaa A. , Physiological Changes in Pregnancy: Review Articles, Cardiovascular journal of Africa. (2016) 27, no. 2, 89–94, 10.5830/CVJA-2016-021, 2-s2.0-84973470248. 27213856 PMC4928162

[11] Mazughi I. , Arebi A. , and Sherif F. M. , Prevalence of Anemia Among Libyan Pregnant Women and Its Relation to Low Birth Weight, Prevalence. (2018) 2, no. 12, 1–6.

[12] World Health Organization , Global Anaemia Estimates in Women of Reproductive Age, by Pregnancy Status, and in Children Aged 6-59 Months, 2019, https://www.who.int/data/gho/data/themes/topics/anaemia_in_women_and_children?form=MG0AV3.

[13] Mudau M. , Peters R. P. , De Vos L. et al., High Prevalence of Asymptomatic Sexually Transmitted Infections Among Human Immunodeficiency Virus-Infected Pregnant Women in a Low-Income South African Community, International Journal of STD & AIDS. (2018) 29, no. 4, 324–333, 10.1177/0956462417724908, 2-s2.0-85036635070. 28799824 PMC6879095

[14] Ahmed R. H. , Yussuf A. A. , Ali A. A. et al., Anemia Among Pregnant Women in Internally Displaced Camps in Mogadishu, Somalia: A Cross-Sectional Study on Prevalence, Severity and Associated Risk Factors, BMC Pregnancy and Childbirth. (2021) 21, no. 1, 1–9, 10.1186/s12884-021-04269-4. 34906104 PMC8670163

[15] Fite M. B. , Assefa N. , and Mengiste B. , Prevalence and Determinants of Anemia Among Pregnant Women in Sub-Saharan Africa: A Systematic Review and Meta-Analysis, Archives of Public Health. (2021) 79, 1–11, 10.1186/s13690-021-00711-3. 34861892 PMC8643002

[16] World Health Organization , Haemoglobin Concentrations for the Diagnosis of Anaemia and Assessment of Severity, 2011, World Health Organization.

[17] Liyew A. M. , Tesema G. A. , Alamneh T. S. et al., Prevalence and Determinants of Anemia Among Pregnant Women in East Africa; A Multi-Level Analysis of Recent Demographic and Health Surveys, Public Library of Science ONE. (2021) 16, no. 4, 10.1371/journal.pone.0250560. PMC8078763 33905448

[18] Group WB , Prevalence of Anemia in Pregnant Women. World Health Organization, Global Health Observatory Data Repository/World Health Statistics, 2019, World Bank Publications.

[19] Smith C. , Teng F. , Branch E. , Chu S. , and Joseph K. , Maternal and Perinatal Morbidity and Mortality Associated With Anemia in Pregnancy, Obstetrics & Gynecology. (2019) 134, no. 6, 1234–1244, 10.1097/aog.0000000000003557. 31764734 PMC6882541

[20] Tunkyi K. and &Moodley J. , Anemia and Pregnancy Outcomes: A Longitudinal Study, Journal of Maternal-Fetal and Neonatal Medicine. (2018) 31, no. 19, 2594–2598, 10.1080/14767058.2017.1349746, 2-s2.0-85023206235. 28697657

[21] Demmouche A. , Lazrag A. , and Moulessehoul S. , Prevalence of Anaemia in Pregnant Women During the Last Trimester: Consequences for Birth Weight, European Review for Medical and Pharmacological Sciences. (2011) 15, no. 4, 436–445. 21608439

[22] Mahmood T. , Rehman A. U. , Tserenpil G. et al., The Association Between Iron-deficiency Anemia and Adverse Pregnancy Outcomes: A Retrospective Report From Pakistan, Cureus. (2019) 11, no. 10, 10.7759/cureus.5854. PMC6830848 31754588

[23] Lebso M. , Anato A. , and Loha E. , Prevalence of Anemia and Associated Factors Among Pregnant Women in Southern Ethiopia: A Community Based Cross-Sectional Study, Public Library of Science ONE. (2017) 12, no. 12, 10.1371/journal.pone.0188783, 2-s2.0-85038408339. PMC5724831 29228009

[24] Leenstra T. , Kariuki S. K. , Kurtis J. D. , Oloo A. J. , Kager P. A. , and ter Kuile F. O. , Prevalence and Severity of Anemia and Iron Deficiency: Cross-Sectional Studies in Adolescent Schoolgirls in Western Kenya, European Journal of Clinical Nutrition. (2004) 58, no. 4, 681–691, 10.1038/sj.ejcn.1601865, 2-s2.0-16544374708. 15042138

[25] Abdilahi M. M. , Kiruja J. , Farah B. O. et al., Prevalence of Anemia and Associated Factors Among Pregnant Women at Hargeisa Group Hospital, Somaliland, BMC Pregnancy and Childbirth. (2024) 24, no. 1, 10.1186/s12884-024-06539-3. PMC11080199 38724919

[26] Mohamed A. I. , Mohamed J. , Abdillahi M. M. , Abdeeq B. A. , and Lema T. B. , Magnitude and Determinants of Adherence to Iron-Folic Acid Supplementation Among Somaliland Pregnant Women in Ahmed-Dhagah District: A Facility Based Cross-Sectional Study, Clinical Epidemiology and Global Health. (2024) 26, 10.1016/j.cegh.2024.101565.

[27] Development Smoh , The Ministry of Health Development in Republic of Somaliland, 2023, Development Smoh.

[28] Addis Alene K. and &Mohamed Dohe A. , Prevalence of Anemia and Associated Factors Among Pregnant Women in an Urban Area of Eastern Ethiopia, Anemia. (2014) 2014. 10.1155/2014/561567 PMC4158560 25215230

[29] Abriha A. , Yesuf M. E. , and Wassie M. M. , Prevalence and Associated Factors of Anemia Among Pregnant Women of Mekelle Town: A Cross Sectional Study, BioMed Central Research Notes. (2014) 7, no. 1, 1–6, 10.1186/1756-0500-7-888, 2-s2.0-84928814732. 25487251 PMC4295569

[30] Azhar B. S. , Islam M. S. , and Karim M. R. , Prevalence of Anemia and Associated Risk Factors Among Pregnant Women Attending Antenatal Care in Bangladesh: A Cross-Sectional Study, Primary Health Care Research & Development. (2021) 22, 10.1017/s146342362100061x. PMC8569827 34727999

[31] Getahun W. , Belachew T. , and Wolide A. D. , Burden and Associated Factors of Anemia Among Pregnant Women Attending Antenatal Care in Southern Ethiopia: Cross Sectional Study, BioMed Central Research Notes. (2017) 10, no. 1, 1–7, 10.1186/s13104-017-2605-x, 2-s2.0-85023743297. 28705235 PMC5512984

[32] Yeneabat T. , Adugna H. , Asmamaw T. et al., Maternal Dietary Diversity and Micronutrient Adequacy During Pregnancy and Related Factors in East Gojjam Zone, Northwest Ethiopia, 2016, BioMed Central Pregnancy and Childbirth. (2019) 19, no. 1, 1–9, 10.1186/s12884-019-2299-2, 2-s2.0-85066043587. 31092223 PMC6521398

[33] Udho S. , Nankumbi J. , Namutebi M. , Mukunya D. , Ndeezi G. , and Tumwine J. K. , Prevalence of Anaemia in Pregnancy and Associated Factors in Northern Uganda: A Cross-Sectional Study, South African Journal of Clinical Nutrition. (2023) 36, no. 4, 136–141, 10.1080/16070658.2022.2148909.

[34] Dhillon P. , Kaur I. , and Singh K. , Pregnancy-Induced Hypertension: Role of Drug Therapy and Nutrition in the Management of Hypertension, PharmaNutrition. (2021) 15, 10.1016/j.phanu.2021.100251.

[35] Kare A. P. and &Gujo A. B. , Anemia Among Pregnant Women Attending Ante Natal Care Clinic in Adare General Hospital, Southern Ethiopia: Prevalence and Associated Factors, Health Services Insights. (2021) 14, 10.1177/11786329211036303. PMC8327009 34376992

[36] Murphy J. , Haemoglobin Concentrations for the Diagnosis of Anaemia and Assessment of Severity, Vitamin and Mineral Nutrition Information System. (2011) World Health Organization, Geneva.

[37] World Health Organization , Basic Malaria Microscopy, 2010, World Health Organization.

[38] World Health Organization , The State of Food Security and Nutrition in the World 2020: Transforming Food Systems for Affordable Healthy Diets, 2020, Food & Agriculture Org.

[39] Ahmed S. , Al Mamun M. A. , Mahmud N. et al., Prevalence and Associated Factors of Anemia Among Pregnant Women Receiving Antenatal Care (ANC) at Fatima Hospital in Jashore, Bangladesh: A Cross-Sectional Study, Food and Nutrition Sciences. (2019) 10, no. 9, 1056–1071, 10.4236/fns.2019.109076.

[40] Majed N. , Alkaseh A. , Ahmed K. , Albrrane E. , and Suliman A. , Prevalence of Anemia and Associated Risk Factor Among Pregnant Women in Al Bayda City-Libya, AlQalam Journal of Medical and Applied Sciences. (2023) 12, 305–312.

[41] Okube O. T. , Mirie W. , Odhiambo E. , Sabina W. , and Habtu M. , Prevalence and Factors Associated With Anaemia Among Pregnant Women Attending Antenatal Clinic in the Second and Third Trimesters at Pumwani Maternity Hospital, Kenya, Open Journal of Obstetrics and Gynecology. (2016) 6, no. 1, 16–27, 10.4236/ojog.2016.61003.

[42] Maktari L. , Naggar R. , and Jaber N. , Prevalence and Associated Factors Among Pregnant Woman With Anemia in Al-Ahawra Hospital Sana’A City Yemen, Hematol Blood Disord. (2021) 4, no. 5, 6–11.

[43] Suryanarayana R. , Chandrappa M. , Santhuram A. N. , Prathima S. , and Sheela S. , Prospective Study on Prevalence of Anemia of Pregnant Women and Its Outcome: A Community Based Study, Journal of Family Medicine and Primary Care. (2017) 6, no. 4, 10.4103/jfmpc.jfmpc_33_17. PMC5848390 29564255

[44] Dahie H. A. and & Heyle A. A. , Prevalence of Anemia and Its Associated Factors Among Pregnant Women Attending Antenatal Clinic at SOS Hospital in Heliwa District, Mogadishu, Advances in Social Sciences Research Journal. (2017) 4, no. 15, 10.14738/assrj.415.3576.

[45] El-Kholy A. A. , El Kholy E. A. , Abdou A. H. et al., Prevalence and Associated Factors of Anemia Among Pregnant Women and the Impact of Clinical Pharmacist Counseling on Their Awareness Level: A Cross Sectional Study, Saudi Pharmaceutical Journal. (2023) 31, no. 8, 10.1016/j.jsps.2023.101699. PMC10393807 37538193

[46] Lestari S. , Fujiati I. , Keumalasari D. , Daulay M. , Martina S. J. , and Syarifah S. , The Prevalence of Anemia in Pregnant Women and Its Associated Risk Factors in North Sumatera, Indonesia, IOP Conference Series: Earth and Environmental Science. (2018) IOP Publishing.

[47] Wemakor A. , Prevalence and Determinants of Anaemia in Pregnant Women Receiving Antenatal Care at a Tertiary Referral Hospital in Northern Ghana, BioMed Central Pregnancy and Childbirth. (2019) 19, 1–11, 10.1186/s12884-019-2644-5. 31829146 PMC6907326

[48] Omote V. , Ukwamedua H. A. , Bini N. , Kashibu E. , Ubandoma J. R. , and Ranyang A. , Prevalence, Severity, and Correlates of Anaemia in Pregnancy Among Antenatal Attendees in Warri, South-Southern Nigeria: A Cross-Sectional and Hospital-Based Study, Sahel Medical Journal. (2020) 4, no. 18, 182–187, 10.1155/2020/1915231. PMC7232721 32455008

[49] Mohamed N. E. B. and &Hassan R. H. A-A. , Prevalence and Factors Associated With Anemia Among Pregnant Women Attending Ante-Natal Clinic in the Second and Third Trimesters at Soba University Hospital, Open Journal of Obstetrics and Gynecology. (2020) 6.

[50] Elmugabil A. and &Adam I. , Prevalence and Associated Risk Factors for Anemia in Pregnant Women in White Nile State, Sudan: A Cross-Sectional Study, SAGE Open Nursing. (2023) 9, 10.1177/23779608231173287. PMC10161327 37153491

[51] Varghese J. S. , Swaminathan S. , Kurpad A. V. , and Thomas T. , Demand and Supply Factors of Iron-Folic Acid Supplementation and Its Association With Anaemia in North Indian Pregnant Women, Public Library of Science ONE. (2019) 14, no. 1, 10.1371/journal.pone.0210634, 2-s2.0-85060803390. PMC6353128 30699167

[52] Siteti M. C. , Namasaka S. D. , Ariya O. P. , Injete S. D. , and Wanyonyi W. A. , Anaemia in Pregnancy: Prevalence and Possible Risk Factors in Kakamega County, Kenya, Science Journal of Public Health. (2014) 2, no. 3, 216–222, 10.11648/j.sjph.20140203.23.

[53] Nasir B. B. , Fentie A. M. , and Adisu M. K. , Adherence to Iron and Folic Acid Supplementation and Prevalence of Anemia Among Pregnant Women Attending Antenatal Care Clinic at Tikur Anbessa Specialized Hospital, Ethiopia, Public Library of Science ONE. (2020) 15, no. 5, 10.1371/journal.pone.0232625. PMC7197778 32365114

[54] Shill K. B. , Karmakar P. , Kibria M. G. et al., Prevalence of Iron-Deficiency Anaemia Among University Students in Noakhali Region, Bangladesh, Journal of Health, Population and Nutrition. (2014) 32, no. 1, 103–110. 24847599 PMC4089078

[55] Etheredge A. J. , Premji Z. , Gunaratna N. S. et al., Iron Supplementation in iron-replete and Nonanemic Pregnant Women in Tanzania: A Randomized Clinical Trial, Journal of the American Medical Association Pediatrics. (2015) 169, no. 10, 947–955, 10.1001/jamapediatrics.2015.1480, 2-s2.0-84943373093. 26280534 PMC4904713

[56] Peña‐Rosas J. P. and &Viteri F. E. , Effects and Safety of Preventive Oral Iron or Iron+ Folic Acid Supplementation for Women During Pregnancy, Cochrane Database of Systematic Reviews. (2009) 4. 10.1002/14651858.CD004736.pub3 19821332

[57] Dodzo R. , Ogunsakin R. E. , and Ginindza T. G. , Prevalence and Associated Risk Factors for Anaemia Among Pregnant Women Attending Ante-Natal Clinic, African Journal of Primary Health Care & Family Medicine. (2021) 14, no. 1. 10.4102/phcfm.v14i1.3339 PMC9082230 35532109

[58] Berhe B. , Mardu F. , Legese H. et al., Prevalence of Anemia and Associated Factors Among Pregnant Women in Adigrat General Hospital, Tigrai, Northern Ethiopia, 2018, BioMed Central Research Notes. (2019) 12, no. 1, 1–6, 10.1186/s13104-019-4347-4, 2-s2.0-85066476843. 31151463 PMC6544916

[59] Townsley D. M. , Hematologic Complications of Pregnancy, Seminars in Hematology. (2013) Elsevier. 10.1053/j.seminhematol.2013.06.004 PMC3748382 23953339

[60] Girma S. , Teshome T. , Worku M. et al., Anemia and Associated Factors Among Pregnant Women Attending Antenatal Care at Madda Walabu University Goba Referral Hospital, Bale Zone, Southeast Ethiopia, Journal of Blood Medicine. (2020) 11, 479–485, 10.2147/jbm.s285190. 33376435 PMC7765680

[61] Tegegne K. T. , Tegegne E. T. , Tessema M. K. , and Ayalew A. , Does Intestinal Parasite Infection Causes Anemia Among Pregnant Women in Ethiopia, Biomedical Journal of Scientific & Technical Research. (2021) 38.

[62] Sapkota L. and &Maharjan M. , Anaemia Association With Intestinal Parasitic Infection in Pregnant Women Attending Antenatal Clinic at Tribhuvan University Teaching Hospital, Journal of Advanced College of Engineering and Management. (2017) 3, 41–47, 10.3126/jacem.v3i0.18885.

[63] Ejeta E. , Alemnew B. , Fikadu A. et al., Prevalence of Anaemia in Pregnant Womens and Associated Risk Factors in Western Ethiopia, Food Science and Quality Management. (2014) 31, no. 6.

[64] Sumampouw O. J. , Nelwan J. E. , and Rumayar A. A. , Outline Images Download Cite Share Favorites Permissions Original Article Socioeconomic Factors Associated With Diarrhea Among Under-Five Children in Manado Coastal Area, Indonesia, Journal of Global Infectious Diseases. (2019) 21, 140–161. 10.4103/jgid.jgid_105_18 PMC6906894 31849434

[65] Chua C. L. L. , Khoo S. K. M. , Ong J. L. E. , Ramireddi G. K. , Yeo T. W. , and Teo A. , Malaria in Pregnancy: From Placental Infection to Its Abnormal Development and Damage, Frontiers in Microbiology. (2021) 12, 10.3389/fmicb.2021.777343. PMC8636035 34867919

[66] Yesuf N. N. and & Agegniche Z. , Prevalence and Associated Factors of Anemia Among Pregnant Women Attending Antenatal Care at Felegehiwot Referral Hospital, Bahirdar City: Institutional Based Cross-Sectional Study, International Journal of Africa Nursing Sciences. (2021) 15, 10.1016/j.ijans.2021.100345.

